# Production and application of mouse monoclonal antibodies targeting linear epitopes in pB602L of African swine fever virus

**DOI:** 10.1007/s00705-021-05335-0

**Published:** 2022-01-05

**Authors:** Pengfei Wang, Chunguo Liu, Shida Wang, Lili Wen, Zhibin Shi, Yue Chi, Ming Wang, Zaisi Liu, Zhenzhao Sun, Lili Wei, Decheng Yang, Xijun He, Jingfei Wang

**Affiliations:** State Key Laboratory of Veterinary Biotechnology, Harbin Veterinary Research Institute of Chinese Academy of Agricultural Sciences, Harbin, 150069 China

## Abstract

African swine fever (ASF) is an acute hemorrhagic disease of domestic pigs. The causative agent of ASF, ASF virus (ASFV), is a double-stranded DNA virus, the sole member in the family *Asfarviridae*. The non-structural protein pB602L of ASFV is a molecular chaperone of the major capsid protein p72 and plays a key role in icosahedral capsid assembly. This protein is antigenic and is a target for developing diagnostic tools for ASF. To generate monoclonal antibodies (mAbs) against pB602L, a prokaryotically expressed recombinant pB602L protein was produced, purified, and used as an antigen to immunize mice. A total of eight mouse mAbs were obtained, and their binding epitopes were screened by Western blot using an overlapping set of polypeptides from pB602L. Three linear epitopes were identified and designated epitope 1 (^366^ANRERYNY^373^), epitope 2 (^415^GPDAPGLSI^423^), and epitope 3 (^498^EMLNVPDD^505^). Based on the epitope recognized, the eight mAbs were placed into three groups: group 1 (B2A1, B2F1, and B2D10), group 2 (B2H10, B2B2, B2D8, and B2A3), and group 3 (B2E12). The mAbs B2A1, B2H10, and B2E12, each representing one of the groups, were used to detect pB602L in ASFV-infected porcine alveolar macrophages (PAMs) and pig tissues, using an indirect fluorescence assay (IFA) and immunohistochemical staining, respectively. The results showed that pB602L was detectable with all three mAbs in immunohistochemical staining, but only B2H10 was suitable for detecting the proteins in ASFV-infected PAMs by IFA. In summary, we developed eight anti-pB602L mouse mAbs recognizing three linear epitopes in the protein, which can be used as reagents for basic and applied research on ASFV.

## Introduction

African swine fever (ASF) is an acute hemorrhagic disease of domestic pigs. This disease often results in devastating economic losses to the pig industry of affected countries because of its high rate of morbidity and mortality, and it is therefore listed as a notifiable disease by the World Organization for Animal Health (OIE). An effective vaccine is still not available, and control of the disease relies mainly on rapid diagnosis and culling of infected pigs and preventing close contact with them [20].

ASF was first recognized in the early 1900s in East Africa. Since then, it has spread to most sub-Saharan African countries [4]. However, after the disease was introduced into Georgia in the Caucasus in 2007 [20], it spread toward the northern and eastern areas of Europe, affecting many countries, including the Russian Federation, Ukraine, and Poland [10, 13, 22]. In 2018, the first ASF outbreak was reported in Liaoning Province in China [11, 28]. To date, the disease has occurred in almost every major pig-production area of China [25]. Recent surveillance has identified naturally attenuated ASFV strains in several provinces of China, making the control situation even more complex [23]. The disease has also been reported recently in other Asian countries [17].

ASF virus (ASFV) is the only member of the family *Asfarviridae* [2]. An ASFV virion has an overall icosahedral morphology with a diameter of 260-300 nm. It is composed of five layers, including a viral core, a core shell, an inner lipid membrane, an icosahedral capsid, and an external envelope [26]. The viral genome is a double-stranded DNA molecule of 170-190 kbp, encoding more than 150 viral proteins. About 50 of the viral proteins are structural proteins with important functions in the viral life cycle, such as viral particle assembly and infection of the host cell. The rest of them are non-structural proteins that are expressed during viral replication and function mainly as regulators of replication. The functions of about 40 proteins of ASFV are still unknown [1].

The pB602L protein of ASFV is encoded by the B602L gene, which contains a central variable region (CVR) and is frequently used for subgenotype classification of ASFV isolates [3, 8, 16, 18]. pB602L is a non-structural protein that functions as a molecular chaperon of the major structural protein p72, which forms aberrant "zipper-like" structures instead of icosahedral virus particles in the absence of pB602L. However, it is not understood how pB602L helps p72 to assemble correctly. Inhibition of the synthesis of pB602L affects the proteolytic processing of two viral polyproteins of ASFV, pp220 and pp62, leading to a decrease in the production of p72 and delocalization of the capsid protein pE120R [7, 15]. Previous studies have shown that pB602L is strongly antigenic and can be used to develop diagnostic tools for ASFV. Previously, a pB602L-based ELISA assay was employed to detect serum antibodies against ASFV, and the test results were mostly consistent with those obtained using the gold-standard Western blot test. Furthermore, this assay was capable of distinguishing pigs that were persistently infected with the natural ASFV strains from those immunized with structural-protein-based subunit vaccines [14]. Given that live-attenuated ASFV strains have shown the most promise as vaccines against ASFV [5, 21], pB602L is probability a suitable target for developing diagnostic tools for evaluating the humoral immune responses of these vaccines, because antibodies against pB602L are produced only after this protein is expressed in host cells. However, the molecular basis for the antigenicity of pB602l remains unclear, and specific mAbs against this protein are still unavailable, which has restrained both applied and basic research on pB602L, including the development of a competition ELISA assay and an anti-ASFV mAb drug.

In this study, we expressed and purified a recombinant pB602L of ASFV strain HLJ/2018, which was then used as an antigen to immunize mice for monoclonal antibody (mAb) production. A total of eight mAbs were obtained, and they were found to bind to three linear epitopes in pB602L. This is the first report on mAbs against linear epitopes of pB602L. These results provide biological materials and a molecular basis for basic and applied research on ASFV.

## Materials and methods

### Ethics statement

The animal experiment with mice was approved by the Animal Care and Use Committee of Harbin Veterinary Research Institute (ID: HVRI-IACUC-2019-348), and the experimental procedure was carried out in strict accordance with the recommendations in the Guide for the Care and Use of Laboratory Animals of the Ministry of Science and Technology of the People’s Republic of China.

### Cells, serum samples, and experimental animals

SP2/0 cells were grown in DMEM medium (Gibco, USA) supplemented with 10% fetal bovine serum (FBS) at 37 °C with 5% CO_2_. Sf-21 cells were grown in Sf-900 II SFM medium (Gibco, USA) at 27 °C with shaking at 120 rpm. Porcine alveolar macrophages (PAMs) infected with ASFV (Pig/HLJ/2018), tissues (spleens, tonsils, and gastrohepatic lymph nodes) collected from ASFV-infected pigs, and anti-ASFV-positive sera were obtained from the National High Containment Facilities for Animal Diseases Control and Prevention, Harbin, China. The details of animal experiments were described previously [5, 27]. Six-week-old female BALB/c mice used in this study were supplied by the Laboratory Animal Center of Harbin Veterinary Research Institute.

### Expression and purification of the pB602L protein in *Escherichia coli*

The B602L gene of ASFV (Pig/HLJ/2018) was synthesized and cloned into the pGEX-6P-1-GST vector by GenScript Biotechnology Co., Ltd (Nanjing, Jiangsu, China). The recombinant plasmid pGEX-6P-1-GST-B602L was introduced by transformation into *E. coli* BL21(DE3) (QIAGEN, Hilden, Germany). The cells were cultured in LB medium containing 100 μg of ampicillin per mL at 37 °C with a shaking speed of 220 rpm. When the optical density at 600 nm (OD_600_) reached 0.5-0.6, protein expression was induced by adding 0.3 mM isopropyl β-D-thiogalactoside (IPTG) at 18 °C for 16 h. The cells were lysed using an ultrasonic cell crusher (Sonics, USA) and then centrifuged at 14,000 × *g* for 40 min. Glutathione Sepharose 4B resin (GE Healthcare, Uppsala, Sweden) was used to purify the GST-tagged pB602L protein, which was then treated with PreScission Protease to remove the GST tag. Purified pB602L protein was obtained by passage through Resource Q anion-exchange resin (GE Healthcare, Uppsala, Sweden) and identified using SDS-PAGE and Western blot assays.

### Expression of pB602L protein in an insect cell system

The B602L gene of ASFV (Pig/HLJ/2018) was cloned into the pFastBac-HTA vector by GenScript Biotechnology Co., Ltd (Nanjing, Jiangsu, China). The recombinant plasmid pFastBac-HTA-B602L was introduced by transformation into *E. coli* DH10 Bac (WEIDI, Shanghai, China), which was grown on LB plates containing 50 μg of kanamycin, 7 μg of gentamicin, 10 μg of tetracycline, 100 μg of X-gal, and 40 μg of IPTG per mL at 37 ℃ for 48 h. A white colony was picked and cultured in LB medium containing 50 μg of kanamycin, 7 μg of gentamicin, and 10 μg of tetracycline per mL at 37 ℃ with a shaking speed of 220 rpm for 24 h. The recombinant bacmid was extracted using a DNA isolation kit (Omega, USA) and used to transfect a 25-mL culture of Sf-21 insect cells at a density of 2.5 × 10^6^ cells/mL. Rescued recombinant baculovirus was used to infect Sf-21 cells for pB602L expression, which was verified using an IFA assay.

### Production of monoclonal antibodies against the pB602L protein

A dose of 100 μg of prokaryotically expressed pB602L mixed with an equal volume of complete Freund’s adjuvant or incomplete Freund’s adjuvant (Sigma, USA) was used to immunize mice. Four six-week-old female BALB/c mice were immunized four times at 14-day intervals. Three days after the fourth immunization, spleen cells were harvested and fused with SP2/0 cells. The fused cells were cultured in DMEM medium containing HAT Supplement (Sigma, USA) and 20% FBS. After 10 days, the supernatant of the fused cells was collected, and antibodies against pB602L were detected using an indirect ELISA (iELISA). Positive clones were selected and subcloned three times by limiting dilution. Monoclonal cells were cultured in DMEM medium containing 20% FBS and 1% penicillin-streptomycin. Ten-week-old female BALB/c mice were used for the preparation of ascites containing antibodies against the pB602L protein. The antibody titers in the ascites were determined using iELISA. An SBA Clonotyping System-HRP kit (Southern Biotech, USA) was used to identify the subtypes of the monoclonal antibodies. The specificity of the monoclonal antibodies against the recombinant protein and virions was evaluated using Western blot and IFA.

### Expression of pB602L polypeptide fragments

**Fig. 1:**
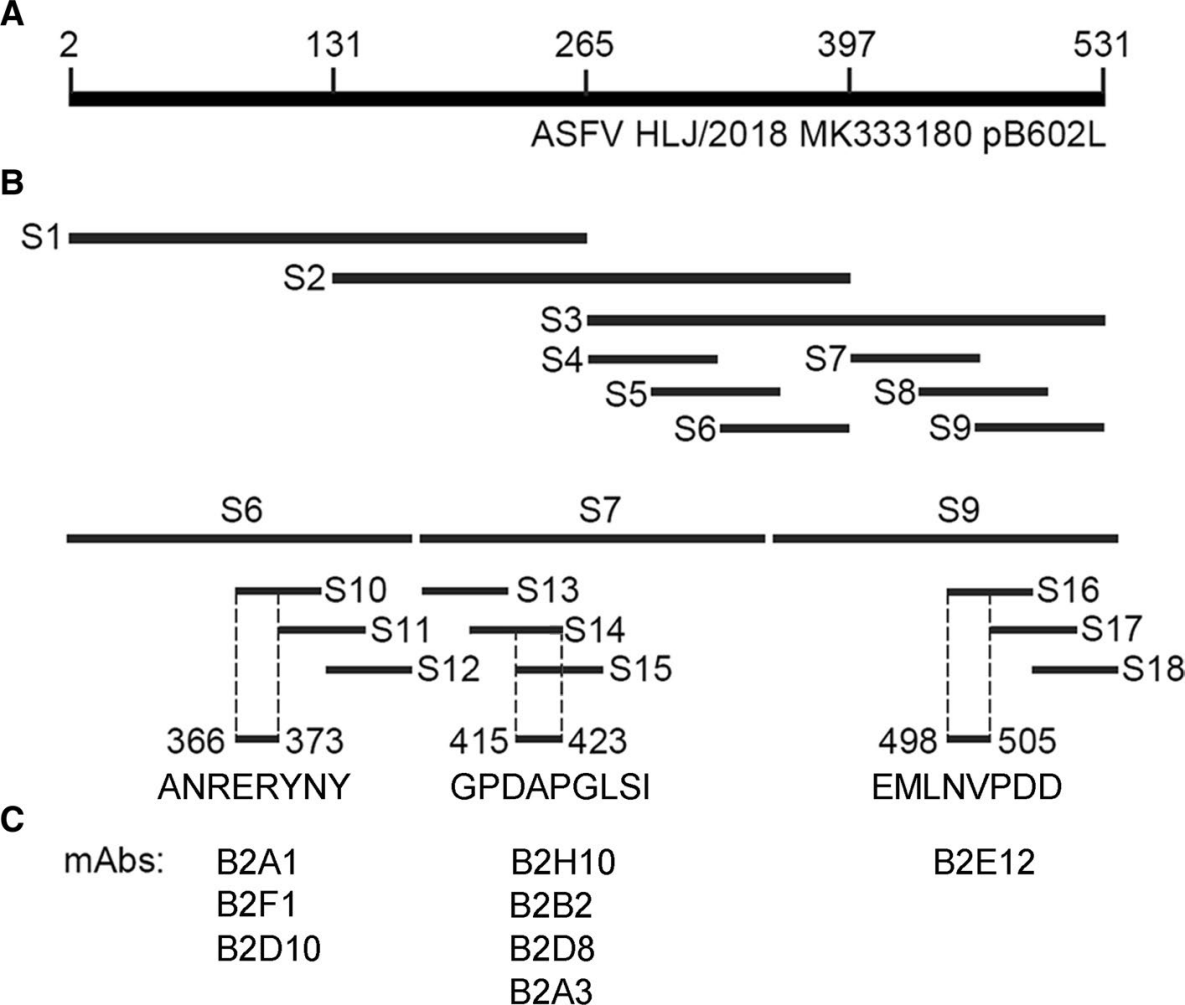
Schematic diagram of epitopes recognized by the mAbs. (**A**) Full-length pB602L protein of ASFV strain Pig/HLJ/2018. (**B**) Design of polypeptide fragments (S1-S18) in pB602L protein and the position of the three epitopes (^366^ANRERYNY^373^, ^415^GPDAPGLSI^423^, ^498^EMLNVPDD^505^). (**C**) List of mAbs reacting with the corresponding pB602L regions

A progressive procedure was adopted to identify the epitope recognized by each mAb, using Western blot assays that were developed based on incrementally shortened pB602L polypeptide fragments. A total of 18 polypeptides (S1-S18) were designed and screened, and the length and position of these polypeptides in pB602L are shown in Fig. 1A and B. Gene fragments encoding polypeptides S1-S9 were amplified from the recombinant plasmid pGEX-6P-B602L template using the primers shown in Table 1. These gene fragments were inserted into plasmid pGEX-6P-1 and introduced by transformation into *E. coli* BL21 (DE3) for expression. The gene fragments encoding polypeptides S10-S18 were synthesized and cloned into the pGEX-6P-1 vector by GenScript Biotechnology Co., Ltd (Nanjing, Jiangsu, China). The procedure for production of these polypeptides was the same as the one described above for the expression of the full-length pB602L protein.

**Table 1:** Primers for amplifying linear plasmid pGEX-6P-1 and gene fragments encoding the polypeptides S1-S9

Primer	Sequence	Target fragment length (bp)
pGEX-6P-1-F	GACTCGAGCGGCCGCAT	4965
pGEX-6P-1-R	TCCCAGGGGCCCCTG	
S1-F	CAGGGGCCCCTGGGAGCGGAGTTCAACATC	792
S1-R	ATGCGGCCGCTCGAGTCTTACGCACGCGGGTT	
S2-F	CAGGGGCCCCTGGGAAAAGAAGCGAAAACC	795
S2-R	ATGCGGCCGCTCGAGTCTTATTCTTTACCGATCTT	
S3-F	CAGGGGCCCCTGGGATTCAAGCCGATCCTG	798
S3-R	ATGCGGCCGCTCGAGTCTTACAGCTCCGCCTT	
S4-F	CAGGGGCCCCTGGGATTCAAGCCGATCCTG	201
S4-R	ATGCGGCCGCTCGAGTCTTACTGGTTGCTGCT	
S5-F	CAGGGGCCCCTGGGACTACGAGGAACTGCG	198
S5-R	ATGCGGCCGCTCGAGTCTTACTTGAAGCTGCCC	
S6-F	CAGGGGCCCCTGGGACAGAAAGTTGACGAG	195
S6-R	ATGCGGCCGCTCGAGTCTTATTCTTTACCGATCTT	
S7-F	CAGGGGCCCCTGGGAATCCTGCGTAACACCATC	198
S7-R	ATGCGGCCGCTCGAGTCTTAATCGTCGATGAA	
S8-F	CAGGGGCCCCTGGGACGGTATCAAGGGCCT	198
S8-R	ATGCGGCCGCTCGAGTCTTATTCCAGAGCGTTA	
S9-F	CAGGGGCCCCTGGGATGCGAGGAAAAGATC	201
S9-R	ATGCGGCCGCTCGAGTCTTACAGCTCCGCCTT	

### SDS-PAGE and Western blot

The samples, including empty-vector-transfected cells, uninduced cells, induced cells, purified pB602L, and polypeptides, were mixed with loading buffer and boiled for 10 min. The proteins in each sample were separated by 10% SDS-PAGE and stained with Coomassie brilliant blue R-250 (Amresco, USA). For Western blot analysis, protein samples were transferred to a PVDF membrane (Thermo, USA) after electrophoresis. The membrane was blocked with 5% Difco skim milk for 2 h at room temperature and then incubated with one of the mAbs or a mouse monoclonal anti-GST antibody (Sigma-Aldrich, USA) (1:2,000) as the primary antibody for 1 h at room temperature. After four washes with Tris-buffered saline containing 0.5% Tween-20 (TBST), the membrane was incubated with IRDye 800CW goat anti-mouse IgG (H + L) as the secondary antibody (LI-COR, USA) (1:5,000) at room temperature for 1 h, followed by four washes with TBST. The blot was analyzed by scanning, using an Odyssey CLx Imaging System (LI-COR, USA).

### Indirect ELISA

Indirect ELISA was performed as described previously [12]. In brief, purified recombinant pB602L protein diluted in Tris-HCl buffer (pH 8.5) to a final concentration of 2.5 μg/mL was coated onto 96-well ELISA plates (100 μl/well) and incubated overnight at 4 ℃. The supernatant was discarded, and the plate was washed three times with phosphate-buffered saline (PBS) (pH 7.2-7.4) containing 0.5% Tween-20 (PBST). After blocking with 5% Difco skim milk for 1 h at room temperature, the monoclonal antibodies purified from ascites were added to the ELISA plate (100 μl/well), which was incubated at 37 ℃ for 30 min. After six washes with PBST, a rabbit anti-mouse IgG horseradish peroxidase (HRP) conjugate (whole molecule) (Sigma-Aldrich, USA) was added as a secondary antibody at a dilution of 1:2000 and incubated at 37 ℃ for 30 min, followed by six washes with PBST. TMB substrate solution (TianGen Biotech, China) was added to the plate (100 μl/well), which was incubated in the dark at room temperature for 20 min. The reaction was terminated by addition of 2M H_2_SO_4_ (50 μl/well), and the optical density was measured at 450 nm using a microplate reader (ELx808, BioTek, USA).

### Immunofluorescence assay (IFA)

pB602L-expressing Sf-21 cells or PAMs infected with ASFV were fixed with 4% paraformaldehyde. After washing three times with PBS, the cells were blocked with 5% BSA at 37 ℃ for 1 h. The cells were then washed with PBS another three times and inoculated with mAbs (1:1000) or murine anti-pB602L serum at room temperature for 1 h, followed by four washes with PBST. Goat anti-mouse IgG (H+L) Alexa Fluor 488 conjugate (Invitrogen, USA) was added at a dilution of 1:2000, and the cells were incubated at 37 ℃ for 30 min. Images were captured using an inverted fluorescence microscope (EVOS FL, Life, USA).

### Immunohistochemical analysis

For immunohistochemical analysis, tissue samples (including spleens, tonsils, and gastrohepatic lymph nodes) were collected from ASFV-infected pigs and fixed in 10% formalin for 24 h at room temperature. Slides were prepared using a standard procedure for dehydration, embedding, and sectioning. The slides were dried in an oven at 56 ℃ for 15 min and incubated with 3% perhydrol liquid at room temperature, blocked with 5% Difco skim milk for 1 h at room temperature, and then incubated with mAbs at a dilution of 1:100 at 4 ℃ overnight. After five washes with TBST, the slides were incubated with a goat anti-mouse IgG HRP conjugate (Invitrogen, USA) (1:100) for 30 min at 37 ℃, stained with ST hematoxylin (Leica, Germany), and viewed using a DFC550 microscope (Leica, Germany).

### Epitope sequence analysis

A total of 69 full-length pB602L protein sequences of ASFV isolates were obtained from the GenBank database (https://www.ncbi.nlm.nih.gov/). These sequences were aligned using ClustalW, implemented in MEGA 6.06 [24].

## Results

### Preparation of recombinant pB602L

**Fig. 2:**
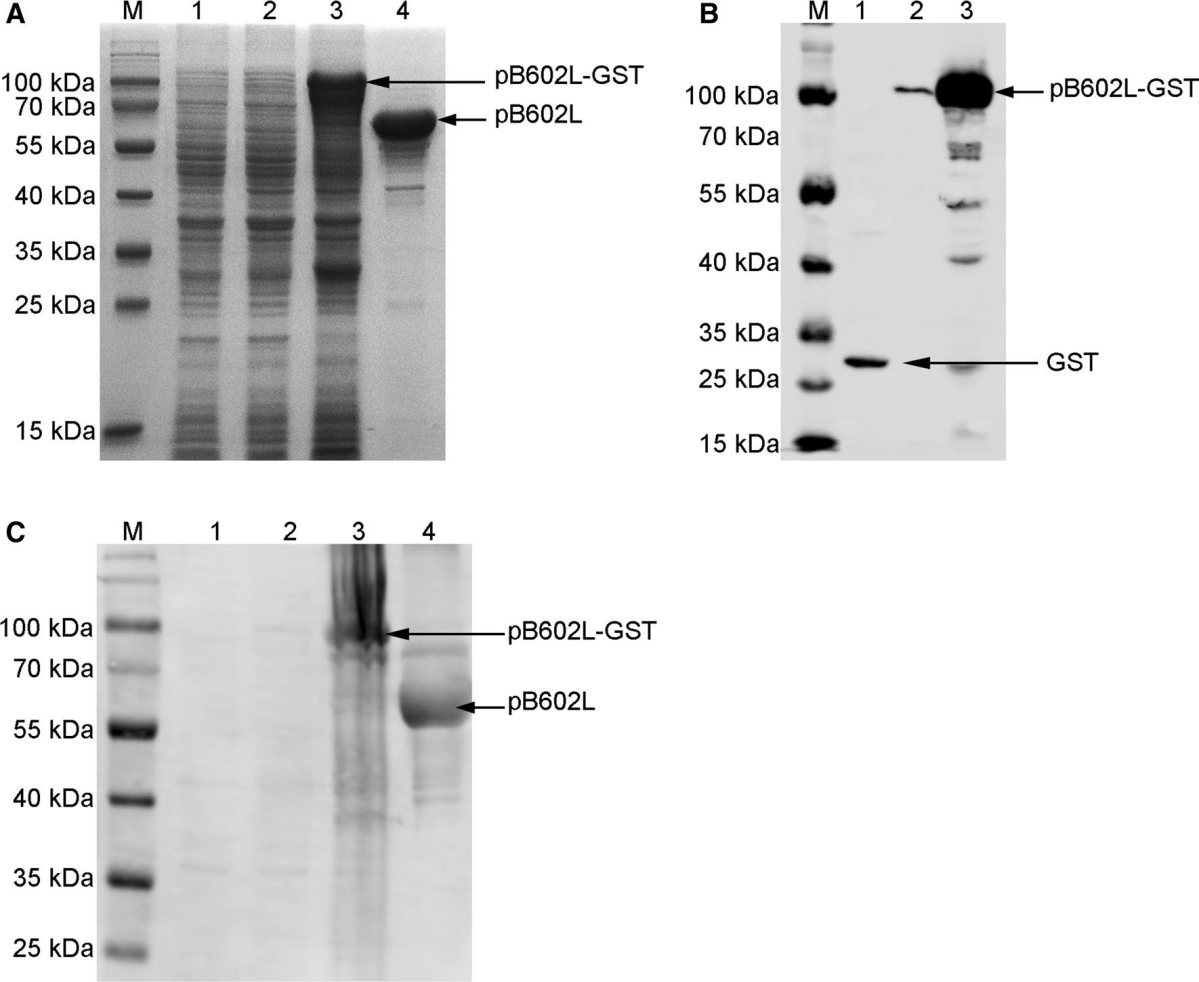
Expression and purification of pB602L. (**A**) SDS-PAGE analysis of pB602L expressed in *E. coli*. (**B**) Identification of pB602L using an anti-GST antibody. (**C**) Identification of pB602L using an anti-ASFV serum. M, protein molecular weight marker; lane 1, cells transfected the empty plasmid pGEX-6P-1; lane 2, uninduced cells transfected the recombinant plasmid pGEX-6P-B602L; lane 3, total cell lysate prepared from cells induced with 0.3 mM IPTG; lane 4, purified pB602L without GST tag

To prepare purified recombinant pB602L for mouse immunization, the B602L gene of ASFV (Pig/HLJ/2018) was cloned into the vector pGEX-6P-1 and expressed as GST-tagged pB602L protein in *E. coli* BL21(DE3). The expression and purity of the recombinant pB602L protein were first assessed by SDS-PAGE. Specific bands corresponding to GST-pB602L and pB602L were observed (Fig. 2A). The expressed pB602L was then identified by Western blot assays using an anti-GST monoclonal antibody (Fig. 2B) and an anti-ASFV pig serum (Fig. 2C). The two assays gave consistent results. The purity of the final purified recombinant protein preparation was estimated to be 95% (Fig. 2A and C), indicating that it was suitable for immunization of mice.

### Production of mouse anti-pB602L mAbs

To produce anti-pB602L monoclonal antibodies, mice were immunized with the purified recombinant pB602L protein. Following a standard protocol for producing mAbs in mice [19], we produced eight clones of anti-pB602L mAbs, and these were designated as B2A1, B2F1, B2D10, B2H10, B2B2, B2D8, B2A3, and B2E12, respectively. These mAbs were found to belong to two subtypes: IgG1 and IgG2a (Table 2). All of them showed strong reactivity with both prokaryotically and eukaryotically expressed recombinant pB602L proteins, as indicated by Western blot, iELISA, and IFA (Table 2).

**Table 2:** Characteristics of the mAbs

mAbs	Subtype	Activity		
		ELISA^†^	IFA^‡^	WB^†^
B2A1	IgG1	+	+	+
B2F1	IgG1	+	+	+
B2D10	IgG1	+	+	+
B2H10	IgG2a	+	+	+
B2B2	IgG2a	+	+	+
B2D8	IgG2a	+	+	+
B2A3	IgG2a	+	+	+
B2E12	IgG1	+	+	+

^†^Antigen expressed in *E. coli*

^‡^Antigen expressed in Sf-21cells

### Epitope mapping

To map the epitopes in pB602L recognized by the mAbs, we tested the mAbs in a Western blot assay for their ability to bind polypeptide fragments of pB602L as shown in Table 3 and Fig. 1B. In this assay, all of the mAbs recognized the largest polypeptide (aa 2-531) (Fig. 1A). We then tested three polypeptides: S1 (aa 2-256), S2 (aa 131-397), and S3 (aa 266-531). In contrast to S1, which was not recognized by any of the mABs, S3 was recognized by all of them. S2 was recognized by mAbs B2A1, B2F1, and B2D10 (Fig. 1C), and these three were therefore placed together in group 1. Based on these results, six additional polypeptides (S4-9) were expressed and screened. The results showed that the group 1 mAbs recognized only S6 (aa 333-397). S7 (aa 399-464) was recognized by four other mAbs (B2H10, B2B2, B2D8, and B2A3) (Fig. 1C), and these were placed together in group 2. S9 (aa 465-531) was recognized by only one mAb (B2E12) (Fig. 1C), which was placed by itself in group 3. To further map the binding sites recognized by each group of mAbs, we produced the overlapping oligopeptides S10-18, and these were also used in screening assays. Based on the overlaps of the polypeptides recognized by the mAbs in each group, three minimal regions were identified and designated as epitope 1 (^366^ANRERYNY^373^), epitope 2 (^415^GPDAPGLSI^423^), and epitope 3 (^498^EMLNVPDD^505^), which were recognized by mAbs of group 1, 2, and 3, respectively (Table 3, Fig. 1B and C).

**Table 3:** Polypeptides and their reactivity with the mAbs

Polypeptide fragment	Amino acid position	Group 1			Group 2				Group 3
		B2A1	B2F1	B2D10	B2H10	B2B2	B2D8	B2A3	B2E12
S1	2-256	−	−	−	−	−	−	−	−
S2	131-397	+	+	+	−	−		−	−
S3	266-531	+	+	+	+	+	+	+	+
S4	266-332	−	−	−	−	−	−	−	−
S5	300-365	−	−	−	−	−	−	−	−
S6	333-397	+	+	+	−	−	−	−	−
S7	399-464	−	−	−	+	+	+	+	−
S8	433-498	−	−	−	−	−	−	−	−
S9	465-531	−	−	−	−	−	−	−	+
S10	366-381	+	+	+	−	−	−	−	−
S11	374-389	−	−	−	−	−	−	−	−
S12	382-397	−	−	−	−	−	−	−	−
S13	398-414	−	−	−	−	−	−	−	−
S14	407-423	−	−	−	+	+	+	+	−
S15	415-432	−	−	−	+	+	+	+	−
S16	498-514	−	−	−	−	−	−	−	+
S17	506-522	−	−	−	−	−	−	−	−
S18	515-531	−	−	−	−	−	−	−	−

Given that the B602L gene has a CVR and that variability is observed among different isolates, we investigated whether these epitopes were conserved among different AFSV isolates by comparing 69 pB602L amino acid sequences downloaded from the GenBank database. The result showed that the positions of the three linear epitopes were located after the CVR and were completely conserved among all of these viruses, suggesting that the mAbs described here have the potential to recognize the pB602L proteins of the other AFSV isolates.

### Preliminary application of the mAbs

**Fig. 3:**
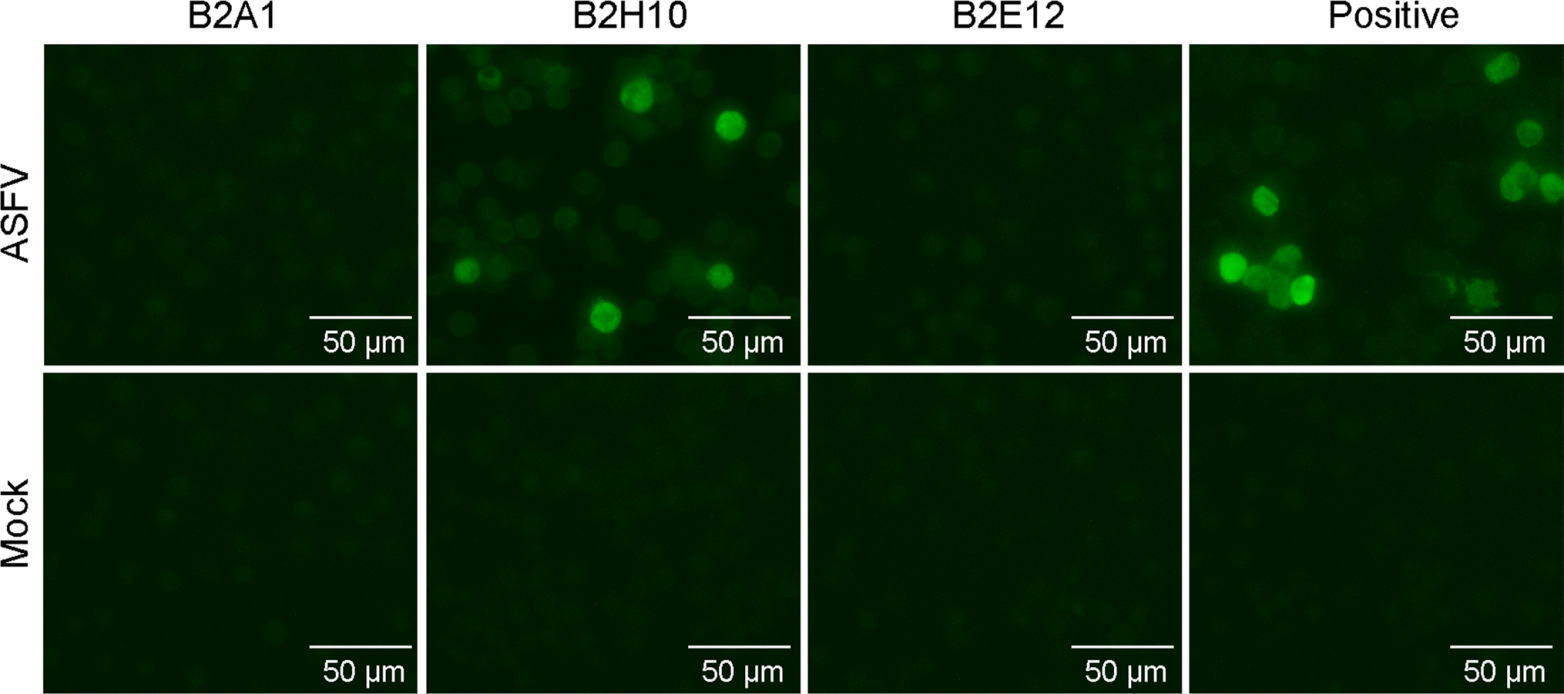
Detection of the pB602L protein using IFA. Primary PAMs infected with ASFV (Pig/HLJ/2018) at an MOI of 0.2 were harvested 24 h postinfection and fixed in 4% paraformaldehyde at 4°C for 24 h, followed by permeabilization with 0.1% (w/v) Triton X-100 at room temperature for 10 min. Representative mAbs of the three groups – B2E12, B2H10, and B2E12 – were incubated with the PAMs infected with ASFV. As a positive control, PAMs were incubated with an anti-porcine ASFV serum. The scale bar represents 50 μm

**Fig. 4:**
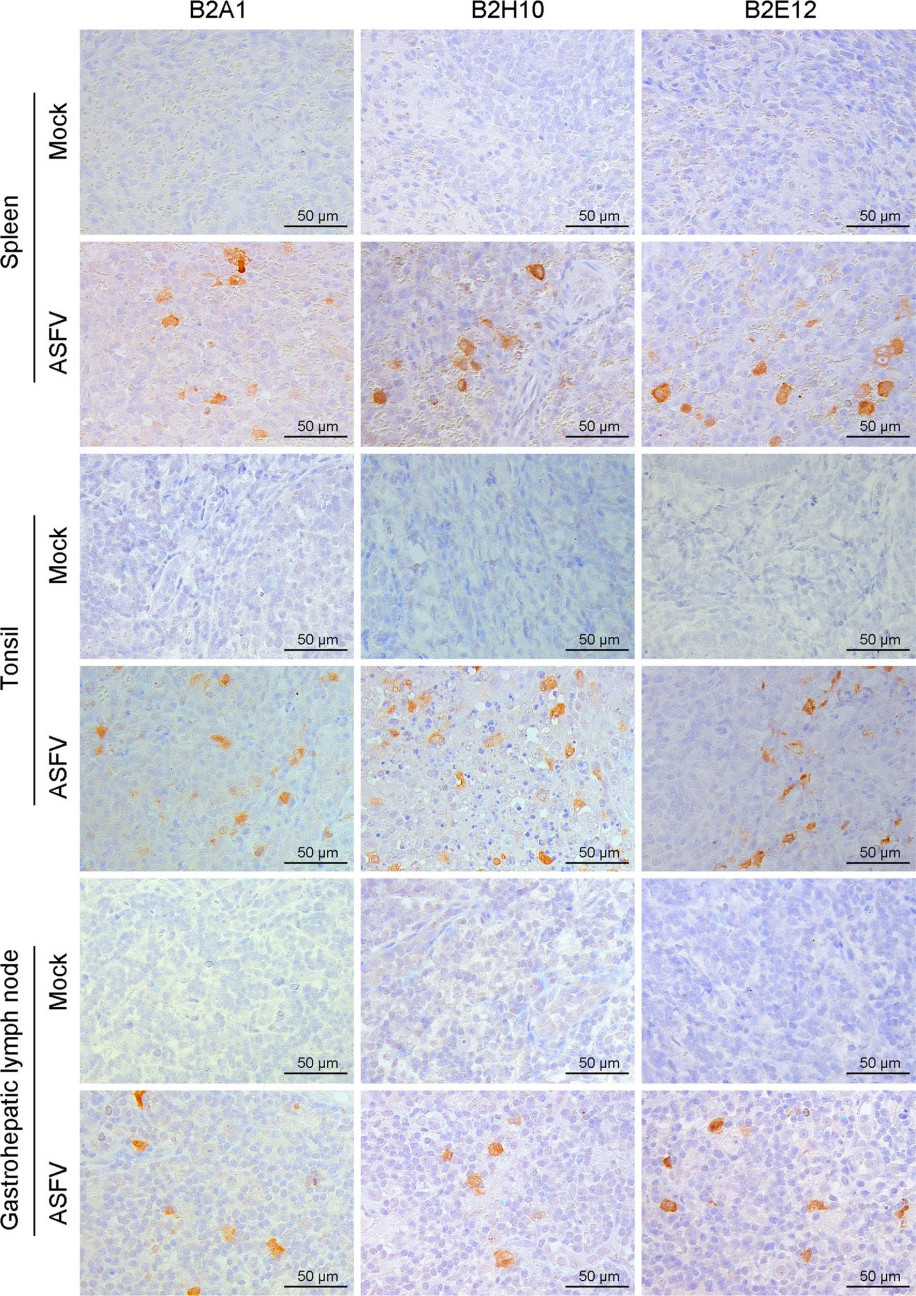
Immunohistochemical assay for detecting pB602L in ASFV-infected pig tissues. Tissues, including the spleens, tonsils, and gastrohepatic lymph nodes, were collected from pigs that were infected with 10^2.5^ times the 50% hemadsorbing dose (HAD_50_) of ASFV strain Pig/HLJ/2018 through intramuscular injection and euthanized on day 7 postinfection. These tissues were fixed, embedded, and sliced using a standard protocol. Three mAbs (B2A1, B2H10, and B2E12) were used as the primary antibodies to stain the pB602L protein in ASFV-infected cells. The scale bar represents 50 μm

To explore the potential application of these mAbs, three mAbs, B2A1, B2H10, and B2E12, representing groups 1, 2, and 3, respectively, were used in IFA, and immunohistochemical staining tests for detecting pB602L in ASFV-infected cells or tissues. During the initial characterization of the mAbs, we determined that all of these mAbs performed well in detecting recombinant pB602L expressed in Sf-21 cells by IFA (data not shown), but when they were used to stain ASFV-infected PAMs, strong fluorescence signals were detected only in the cells stained with B2H10 (Fig. 3). In the immunohistochemical test, antigen-specific brown signals were observed in all of the tissues of the ASFV-infected pigs, including spleen, tonsil, and gastrohepatic lymph node, which were stained with each of the three mAbs, but no staining was observed in the controls (Fig. 4), indicating that all of these mAbs are suitable for immunohistochemical staining.

## Discussion

ASF poses a great threat to the pig industry worldwide. Given there is no effective vaccine available, disease control relies mainly on rapid diagnosis, culling of infected pigs, and enhancing biosecurity measures on the threatened farms in the affected countries [6]. A better understanding of AFSV is key to developing effective control measures. pB602L is an important non-structural protein of ASFV. To date, the few studies that have focused on this protein have shown that it plays an essential role during viral particle assembly and can also be used to develop diagnostic tools [7, 9, 14]. Most of the functions of this protein remain unknown. In the current study, we expressed a recombinant pB602L protein, which was then used to immunize mice for screening mAbs. Eight mouse mAbs were produced, and their epitopes were mapped. To our knowledge, this is the first report on mAbs and epitopes of pB602L, and it provides new knowledge about the molecular basis of the antigenicity of this protein.

mAbs against a viral protein are important biological materials for both basic and applied research on the virus. Previous studies have shown that the antibodies against pB602L were detectable as early as 10 days postinfection in pigs, and ELISA assays developed based on recombinant pB602L proteins have shown performance similar to that of the structural proteins p30 or p54, suggesting that pB602L has good antigenicity and is suitable for developing diagnostic tools [9, 14]. However, the molecular basis for pB602L antigenicity remains largely unknown. Therefore, in this study, we produced eight mouse anti-pB602L mAbs. All of them reacted well with recombinant pB602L. We then identified three epitopes (^366^ANRERYNY^373^, ^415^GPDAPGLSI^423^, and ^498^EMLNVPDD^505^) recognized by these mAbs in pB602L. A multiple sequence alignment based on 69 pB602L amino acid sequences showed that all three epitopes were completely conserved among these ASFV isolates, suggesting that the antigenicity of pB602L is probably determined by the conserved regions rather than the CVR. This could partially explain, despite the presence of a CVR in the B602L gene of different ASFV isolates [16], why a pB602L-based ELISA assay worked well for detecting serum antibodies against different ASFV strains [9]. Furthermore, this also indicates that an mAb targeting at any of these epitopes could possibly recognize the pB602L protein of all of the currently identified ASFV strains.

pB602L plays an essential role in successful assembly of the ASFV capsid [15], making this protein a potential target for anti-ASFV drug screening. However, the current knowledge on this protein is very limited, and much work needs to be done to understand its biological characteristics and the mechanism by which it functions as a molecular chaperone of the p72 protein. Visualization of protein expression in ASFV-infected cells is of great importance for such studies. To test the potential of these mAbs for visualizing the pB602L protein in ASFV-infected cells and tissues, three mAbs – B2A1, B2H10, and B2E12 – were used to detect pB602L in ASFV-infected cells and tissues. An IFA assay revealed that B2H10, which recognizes epitope 2, was able to detect pB602L-positive PAMs, but the other two were not. We repeated the IFA three times, and the results consistently showed that only group 2 mAbs are suitable for detecting pB602L in ASFV-infected PAMs by IFA. When these mAbs were employed to detect ASFV-infected cells in different tissues that were collected from ASFV (Pig/HLJ/2018)-infected pigs, all of them performed well with high specificity and efficiency, indicating that all of them are suitable for immunohistochemical staining. Furthermore, we found that all the mAbs have a high affinity for the pB602L protein. Given that this protein plays an essential role in ASFV assembly, we predict that some of these mAbs may be able to inhibit the functions of pB602L and thus interfere with the production of viral particles. Therefore, these mAbs have the potential to be used as anti-ASFV monoclonal antibody drugs. The anti-ASFV potential of these mAbs should be assessed in the future.

## Conclusions

The monoclonal antibodies produced in this study can be used for the development of ASFV detection tools, and the identification of epitopes in pB602L provides new knowledge for understanding the antigenicity of ASFV.
